# 3D Printing in Nasal Reconstruction: Application-Based Evidence on What Works, When, and Why

**DOI:** 10.3390/biomedicines13061434

**Published:** 2025-06-11

**Authors:** Raisa Chowdhury, Nisreen Al-Musaileem, Karanvir S. Raman, Dana Al-Majid, Philip Solomon, Richard Rival

**Affiliations:** 1 Faculty of Medicine and Health Sciences, McGill University, Montreal, QC H3A 0G4, Canada; 2 Kuwait Ministry of Health, Kuwait City 9XCR+8MR, Kuwait; 3 Division of Plastic, Reconstructive and Aesthetic Surgery, McGill University, Montreal, QC H3A 0G4, Canada; 4 Department of Otolaryngology-Head and Neck Surgery, McGill University, Montreal, QC H3A 0G4, Canada; 5 Department of Otolaryngology-Head and Neck Surgery, University of Toronto, Toronto, ON M5S 3H2, Canada

**Keywords:** 3D printing, nasal reconstruction, patient-specific implants, bioprinting, computational modeling, regenerative medicine

## Abstract

**Background:** Nasal reconstruction requires a balance between aesthetic and functional restoration. Recent advances in three-dimensional (3D) printing have introduced new approaches to this field, enabling precise, patient-specific interventions. This review explores the applications, benefits, and challenges of integrating 3D printing in nasal reconstruction. **Methods**: A literature search was conducted using PubMed, Scopus, and Web of Science to identify studies on 3D printing in nasal reconstruction. Peer-reviewed articles and clinical trials were analyzed to assess the impact of 3D-printed models, implants, and bioengineered scaffolds. **Results**: 3D printing facilitates the creation of anatomical models, surgical guides, and implants, enhancing surgical precision and patient outcomes. Techniques such as stereolithography (SLA) and selective laser sintering (SLS) enable high-resolution, biocompatible constructs using materials like polylactic acid, titanium, and hydroxyapatite. Computational fluid dynamics (CFD) tools improve surgical planning by optimizing nasal airflow. Studies show that 3D-printed guides reduce operative time and improve symmetry. Emerging bioprinting techniques integrating autologous cells offer promise for tissue regeneration. **Challenges and Future Directions**: Challenges include high costs, imaging limitations, regulatory hurdles, and limited vascularization in bioprinted constructs. Future research should focus on integrating bioactive materials, artificial intelligence-assisted design, and regulatory standardization. **Conclusions**: 3D printing offers specific advantages in nasal reconstruction, improving precision and outcomes in selected cases. Addressing current limitations through technological and regulatory advancements will further its clinical integration, potentially enhancing reconstructive surgery techniques.

## 1. Introduction

The nose serves as a central component of facial identity and plays a crucial role in respiration, olfaction, and overall facial aesthetics. Its structural integrity is essential for both functional and cosmetic purposes, making nasal reconstruction a field of significant medical and surgical interest. The origins of nasal reconstruction can be traced back to ancient India, where Sushruta documented pioneering techniques in the *Sushruta Samhita* as a response to punitive nasal amputations, laying the groundwork for modern rhinoplasty [1,2]. Over the centuries, nasal reconstruction has evolved from a necessity-driven procedure addressing trauma, congenital anomalies, and oncologic resections to one that also caters to aesthetic concerns influenced by societal and cultural norms.

Despite significant advances in conventional nasal reconstruction techniques, multiple clinical challenges persist with quantifiable impacts on patient outcomes. Post-surgical complication rates for traditional nasal reconstruction remain considerable, with infection rates ranging from 3.6% to 8.2%, revision surgery requirements of 10–25%, and functional airway compromise in 15–30% of cases. These complications translate to decreased patient satisfaction and higher revision burden—two primary Level 1 clinical problems. Acceptable clinical benchmarks established in the literature suggest that infection rates above 5%, revision rates exceeding 15%, and functional impairment in more than 10% of cases represent suboptimal outcomes. Additionally, conventional autologous tissue harvesting is associated with donor-site morbidity in 20–40% of cases, with pain lasting more than three months reported in up to 15% of patients. Validated patient-reported outcome tools, such as the Rhinoplasty Outcome Evaluation (ROE), frequently show satisfaction rates of only 60–75% with traditional techniques below the 80% threshold generally considered acceptable. These specific clinical challenges represent critical areas where technological innovations may improve results beyond established benchmarks.

We therefore define two primary Level 1 clinical problems in nasal reconstruction: (1) patient-reported satisfaction scores consistently falling below accepted thresholds (60–75% vs. desired ≥80%), and (2) elevated revision surgery rates (10–25%) compared to the acceptable benchmark of <15%. These Level 1 outcomes will serve as the foundation for our hierarchical problem-solving framework.

Achieving optimal outcomes in nasal reconstruction requires a meticulous balance between function and aesthetics. Conventional techniques, including cartilage grafting and skin flaps, have significantly advanced over time, yet challenges such as donor-site morbidity, structural stability, and long-term functional integrity persist. Recent innovations in three-dimensional (3D) imaging and printing have introduced notable advancements in the field by enhancing precision in surgical planning and execution. The integration of 3D-printing technology has helped bridge the gap between traditional surgical craftsmanship and modern biomedical engineering, facilitating patient-specific solutions that aim to reduce complications and improve long-term outcomes.

To address the complex decision making involved in selecting and evaluating these technologies, this review uses a hierarchical framework grounded in clinical problem-solving. This approach prioritizes Level 1 outcomes—patient satisfaction and revision burden—as the primary metrics of success. Level 2 includes intermediate surgical factors such as symmetry, functional airway patency, and complication rates, which are causally linked to Level 1 outcomes. Level 3 encompasses technical parameters, such as 3D printer resolution, material composition, and digital modeling accuracy.

This systematic review examines the role of 3D printing in addressing these specific clinical challenges in nasal reconstruction, with three primary objectives: (1) to evaluate whether current 3D printing applications demonstrably reduce revision surgery rates and improve patient satisfaction scores above clinically acceptable thresholds; (2) to assess evidence for decreased donor-site morbidity relative to conventional techniques; and (3) to analyze whether Level 3 technological innovations lead to measurable Level 1 outcomes in aesthetic and functional domains.

By explicitly aligning our review with this hierarchical problem-solving framework, we aim to determine not only whether 3D printing offers technical innovation, but also whether those innovations solve the central clinical problems of nasal reconstruction. This structure allows for critical evaluation of whether advancements at Level 3 (e.g., printing resolution and material properties) translate to improvements at Level 2 (e.g., surgical precision and operative time) that ultimately achieve clinically meaningful benefits at Level 1 (e.g., patient satisfaction and reduced revision rates).

## 2. Methods

This systematic review was conducted according to the Preferred Reporting Items for Systematic Reviews and Meta-Analyses (PRISMA) 2020 guidelines [3].

### 2.1. Information Sources and Search Strategy

A comprehensive literature search was conducted in three electronic databases: PubMed/MEDLINE, Scopus, and Web of Science. The search covered the period from January 2012 to September 2024.

The search strategy combined terms related to nasal reconstruction and 3D-printing technology. The following keywords were used in combination with Boolean operators: (“nasal reconstruction” OR “rhinoplasty” OR “nose reconstruction” OR “nasal defect”) AND (“3D print” OR “three-dimensional print” OR “additive manufacturing” OR “bioprinting” OR “computer-aided design” OR “CAD/CAM” OR “rapid prototyping”). Additional relevant articles were identified through manual searches of reference lists from the included studies.

### 2.2. Eligibility Criteria

Studies were included based on the following criteria:

- Population: Patients undergoing nasal reconstruction.
- Intervention: Application of 3D-printing technology in nasal reconstruction.
- Comparator: Studies with or without comparison to conventional techniques.
- Outcomes: Studies must report or discuss clinical, functional, or aesthetic outcomes, with explicit or inferred links to the two primary Level 1 problems—patient satisfaction and/or revision burden.
- Study design: Primary research articles including case reports, case series, cohort studies, and clinical trials.
- Language: Full text available in English.
- Publication type: Peer-reviewed journal articles.

Exclusion criteria were as follows:

- Review articles, editorials, letters, and conference abstracts.
- Studies focusing exclusively on other facial reconstructions without nasal involvement.
- Studies without clear description of the 3D-printing methodology.
- Animal or in vitro studies without human application.

### 2.3. Study Selection Process

**Figure 1:**
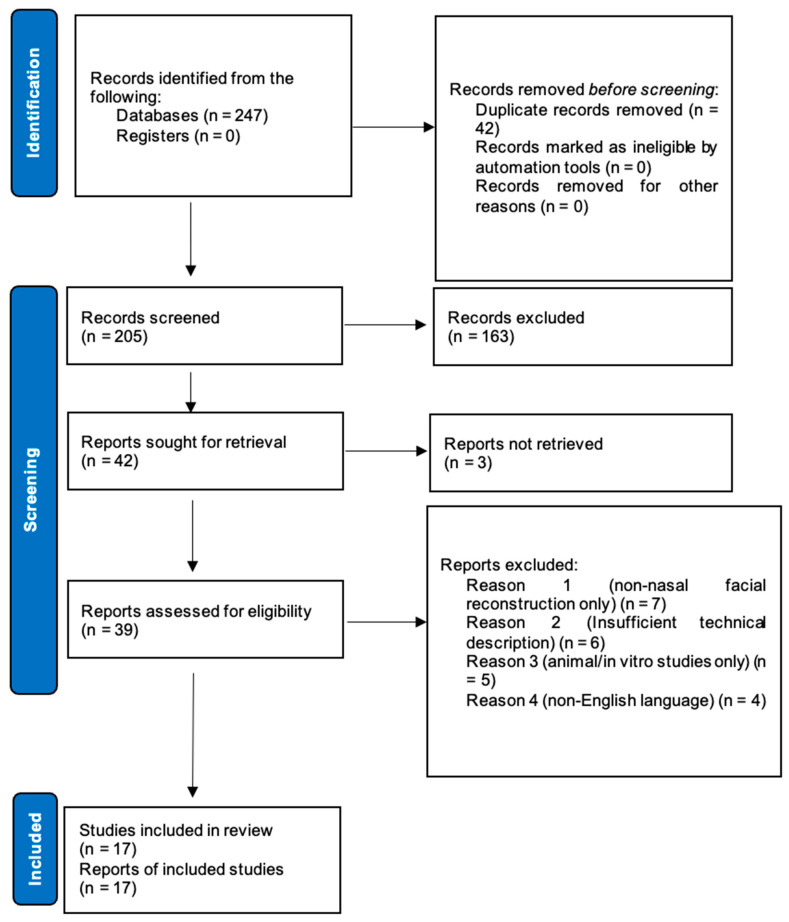
PRISMA flow diagram of study selection process.

After the initial database search, duplicates were removed. Two reviewers independently screened titles and abstracts to identify potentially eligible studies according to the predefined criteria. Full texts of the selected articles were then retrieved and assessed for eligibility. Special attention was paid to whether the study outcomes addressed or could be reasonably connected to Level 1 benchmarks (patient satisfaction ≥80%, revision rates <15%), either directly or through Level 2 surrogate measures (functional, aesthetic, or complication outcomes linked to revisions or satisfaction). Disagreements were resolved through discussion and consensus or, when necessary, consultation with a third reviewer. A PRISMA flow diagram was generated to document the selection process (Figure 1).

### 2.4. Data Extraction

A standardized data extraction form was developed to collect the following information:

- Study characteristics (first author, publication year, study design, and sample size).
- Patient demographics and clinical presentation.
- 3D printing technique and materials.
- Type of 3D-printed product (e.g., surgical guide, implant, and anatomical model).
- Follow-up duration.
- Clinical outcomes relevant to Level 1 (patient satisfaction scores and revision rates) and Level 2 (functional and aesthetic outcomes and complication rates).
- Complications.
- Comparison with conventional techniques when available.

Data extraction was performed independently by two reviewers, with disagreements resolved by consensus or consultation with a third reviewer.

### 2.5. Quality Assessment

The methodological quality of the included studies was assessed based on study design. For case reports and case series, we used the Joanna Briggs Institute (JBI) Critical Appraisal Checklist [4]. For controlled clinical studies, we employed the Cochrane Risk of Bias Tool [5]. Studies were also qualitatively assessed for their ability to inform the core Level 1 clinical questions of patient satisfaction and revision rates, using Level 2 and Level 3 evidence where direct Level 1 outcomes were unavailable. This assessment helped contextualize the strength of evidence when synthesizing findings.

### 2.6. Data Synthesis

Due to the heterogeneity of the included studies in terms of methodologies, patient populations, and outcome measures, a narrative synthesis approach was employed rather than a meta-analysis. The synthesis was explicitly structured according to a hierarchical framework designed to determine whether technical innovations (Level 3) and surgical improvements (Level 2) effectively solved the Level 1 problems of low patient satisfaction and high revision burden, which were defined as the ultimate clinical success criteria.

The synthesis was structured as follows:

- Level 1: Clinical outcomes (patient satisfaction scores, revision rates, functional outcomes, and complication rates).
- Level 2: Intermediate factors (anatomical accuracy, structural stability, and graft integration).
- Level 3: Technical aspects (3D printing methods, materials, and design approaches).

This framework allowed for a systematic evaluation of how advancements in 3D-printing technology translate to meaningful improvements in nasal reconstruction. This hierarchical framework addresses a common limitation in technology assessment: the assumption that statistical improvements in technical parameters automatically translate to clinical benefits. Our analysis explicitly distinguishes between technical capabilities (Level 3), their effect on surgical factors (Level 2), and their ultimate impact on clinical outcomes (Level 1), focusing specifically on whether Level 1 outcomes—patient satisfaction and revision burden—meet or exceed established clinical benchmarks. For instance, a 3D-printed guide with superior dimensional accuracy (Level 3) that reduces operative time (Level 2) only represents meaningful progress if it also improves satisfaction scores or reduces revision rates beyond accepted thresholds (Level 1). This problem-focused approach helps avoid the misconception that impressive technical performance inherently justifies replacing conventional techniques, ensuring our conclusions remain grounded in the most important clinical outcomes.

## 3. Importance of Precise Nasal Reconstruction

Nasal reconstruction requires a precise balance of surgical skill and artistic judgment to restore both function and aesthetics. Successful reconstruction must account for external appearance, skin texture, and color while maintaining natural nasal contours [6]. Achieving a natural contour is critical for facial harmony, as noted in multiple studies [7,8,9]. Ethnically sensitive approaches further enhance outcomes, particularly in diverse populations where auricular composite or costal cartilage grafts provide structural support and better skin tone matching [8,10].

Beyond aesthetics, the nose plays a vital role in respiration, humidification, and olfaction. Structural abnormalities can compromise these functions, emphasizing the need for precise restoration. Rahmawan et al. (2024) highlighted the importance of cartilage grafts in maintaining airway stability and preventing collapse during breathing [9]. Similarly, Strohl et al. (2025) demonstrated that midface reconstructions directly influence airflow dynamics and nasal passage stability [11]. Chrysostomidis et al. (2024) further reported that advanced prosthetics and implants seamlessly integrate with soft tissue, aiding in olfactory function restoration [12].

The nasal subunit principle underscores the need to address distinct tissue characteristics in reconstructive planning, particularly for trauma, congenital anomalies (e.g., cleft lip nasal deformities), and post-oncologic resections. Poor reconstruction can lead to functional deficits, asymmetry, and psychological distress. A 2024 meta-analysis confirmed that nasal reconstruction significantly improves postoperative quality of life across physical, emotional, and social domains [13].

Since the introduction of computer imaging in rhinoplasty in 1983, technological advancements—particularly in 3D printing—have significantly enhanced surgical planning capabilities [14]. 3D-printed patient-specific models allow for more detailed defect assessment, refined reconstructive strategies, and enhanced preoperative simulations, which may improve surgical accuracy and outcomes [15]. This integration of biomedical engineering with reconstructive surgery represents an important development in nasal reconstruction, complementing traditional craftsmanship with modern precision techniques.

The clinical significance of precision in nasal reconstruction is directly linked to specific thresholds that prevent adverse outcomes. However, these precision thresholds (e.g., symmetry, tip projection, and nasal patency) are Level 2 outcomes, which only matter clinically if they ultimately improve Level 1 patient satisfaction or reduce the need for revisions—our two main clinical success metrics. Structural inaccuracies exceeding 2 mm in the nasal framework have been associated with visible external asymmetry and nasal valve compromise [16]. Septal deviation greater than 3 mm from midline increases the risk of functional obstruction, while columellar–labial angle errors exceeding 8° result in patient dissatisfaction in a significant percentage of cases [16].

Thus, these precision thresholds represent concrete clinical targets only when they demonstrably lead to improved satisfaction or fewer revisions. Reconstructive approaches must achieve dimensional accuracy within these tolerances to prevent specific functional and aesthetic complications that ultimately drive revision rates or lower satisfaction. Conventional techniques rely on intraoperative judgment to achieve these targets, with success heavily dependent on surgeon experience. 3D-printing approaches offer the potential to meet these precision requirements more consistently through pre-surgical planning and standardized implementation, particularly in complex cases where multiple geometric relationships must be maintained simultaneously.

However, the clinical value of achieving precision beyond these thresholds remains unproven—there is a point of diminishing returns where additional technical precision no longer translates into meaningful Level 1 clinical improvements, such as patient-reported satisfaction or reduced revision surgeries. For this reason, our hierarchical framework focuses on identifying whether technical or Level 2 surgical improvements actually solve the Level 1 problems rather than assuming all precision advances are equally valuable.

## 4. Advancements in 3D Printing Technology

The development of advanced 3D-printing techniques and biocompatible materials has introduced new approaches to nasal reconstruction. Two key 3D printing methods relevant to this field are stereolithography (SLA) and selective laser sintering (SLS). SLA, one of the earliest 3D-printing technologies, uses ultraviolet light to cure liquid resin layer by layer, producing high-resolution, smooth-surfaced anatomical models and surgical guides. SLS, in contrast, employs a high-powered laser to sinter powdered materials like nylon or polymers into solid structures, making it particularly advantageous for fabricating complex geometries and durable, biocompatible implants. Hybrid approaches such as PolyJet and digital light processing (DLP) are gaining prominence due to their speed and ability to print detailed models using multiple materials, enhancing surgical planning and simulation [17,18]. The key techniques used in 3D printing are listed in Table 1.

Importantly, this section focuses on the Level 3 technical advances, which must be evaluated in terms of whether they ultimately contribute to solving the Level 1 problems (patient satisfaction and revision rates)—not just improving surgical accuracy or intermediate-level outcomes.

The selection criteria incorporate our “Good Enough” principle, indicating when technical specifications are sufficient to achieve clinical goals without unnecessary complexity. For example, while SLA offers superior resolution (25–100 μm), FDM resolution (100–300 μm) is adequate for basic planning models but insufficient for precise surgical guides. Material selection should follow similar threshold-based decisions: general educational models require only basic PLA (FDM), while patient-specific surgical guides demand biocompatible resins (SLA), and load-bearing implants require titanium or reinforced polymers (SLS). This approach promotes resource-appropriate technology selection based on specific clinical needs rather than pursuing technical maximalism.

However, while these technological capabilities represent significant engineering achievements, their clinical value must be assessed through the hierarchical framework to determine whether they meaningfully reduce Level 1 revision rates and improve patient satisfaction—not just deliver Level 2 improvements in surgical accuracy or functional geometry. Advances in resolution from 100 μm to 25 μm with newer SLA printers represent a 75% improvement at the technical level (Level 3), but clinical studies have not demonstrated proportional improvements in patient outcomes (Level 1). Material innovations such as PCL-hydroxyapatite composites show promising in vitro mechanical properties, but their long-term stability in vivo remains unproven beyond 36 months.

The critical clinical relevance of these technical advances lies in whether they address the key bottlenecks that actually drive Level 1 dissatisfaction or the need for revision surgeries. For example, the enhanced resolution of modern SLA printers directly addresses the clinical need for precise surgical guides in complex osteotomies, where conventional templates have shown deviation rates of 15–28%. In contrast, the development of multi-material printing capabilities, while technically impressive, has not yet demonstrated significant improvements in functional outcomes compared to single-material approaches. This distinction reinforces that technological progress should be assessed through application-specific evaluation that explicitly maps Level 3 advances to Level 1 problem solving, not merely assumed as universally beneficial.

Materials used in 3D-printed nasal implants must meet stringent criteria for biocompatibility, structural integrity, and functionality [19,20]. Commonly used polymers include polylactic acid (PLA) and polycaprolactone (PCL), both biodegradable and conducive to tissue integration. PCL, in particular, offers flexibility and durability, making it well-suited for cartilage-regenerating scaffolds [21]. Titanium alloys, fabricated using SLS, are preferred for their strength, corrosion resistance, and biocompatibility, particularly in structural frameworks [22]. The categories of materials used in printing nasal scaffolds and implants are listed in Table 2.

Hydrogels, such as polyethylene glycol diacrylate (PEGDA), and bioactive materials like hydroxyapatite (HA) further enhance nasal reconstruction. HA, a primary component of bone, supports osteointegration and implant stability, making it valuable in reconstructive applications. Similarly, collagen-infused hydrogels facilitate cell attachment and tissue regeneration, ensuring compatibility with natural repair processes. These materials undergo rigorous biocompatibility and durability testing, reinforcing their role in modern nasal reconstruction [17,23].

The integration of biomimetic tissue reconstruction with 3D printing has contributed to the development of 3D bioprinting, where biomaterials, bioactivators, and living cells are used to replicate native tissue architecture. This technology offers enhanced precision and adaptability, potentially expanding possibilities for functional, patient-specific scaffolds in nasal reconstruction [24,25]. However, as with other technical advancements, the ultimate clinical value of bioprinting must be measured by its capacity to achieve acceptable patient satisfaction and reduce revision rates—criteria it has not yet consistently met in clinical settings.

## 5. Applications in Nasal Reconstruction

The integration of 3D printing with advanced imaging technologies such as CT 3D reconstruction, Stereo Photography, Laser Scanning, and Magnetic Resonance Imaging (MRI) has shown potential to improve nasal reconstruction. This section analyzes specific applications organized by type, with each examined through our hierarchical framework linking technical capabilities (Level 3), surgical factors (Level 2), and clinical outcomes (Level 1). It is essential to highlight that only applications showing clear improvements at Level 1—patient satisfaction and revision rates—should be considered evidence of clinical advancement, while others remain promising but unproven at the patient-outcome level.

Furthermore, digital prosthesis design aims to improve aesthetic outcomes and provides a potential alternative for cases with insufficient tissue or vascular compromise. By reducing the need for autologous tissue harvesting, 3D printing may decrease donor-site morbidity, shorten recovery times, and potentially improve patient satisfaction. Clinical case studies summarizing real-world applications of 3D printing in nasal reconstruction are presented in Table 3.

Overall, 3D printing is transforming nasal reconstruction by offering personalized, precise, and functionally superior solutions. When combined with cartilage tissue engineering, these advancements hold immense potential to redefine modern nasal reconstructive surgery. However, as shown in our framework, not all applications have demonstrated measurable Level 1 benefits. The strongest evidence currently exists for surgical guides and patient-specific implants in complex reconstructions where conventional techniques have failed.

**Figure 2:**
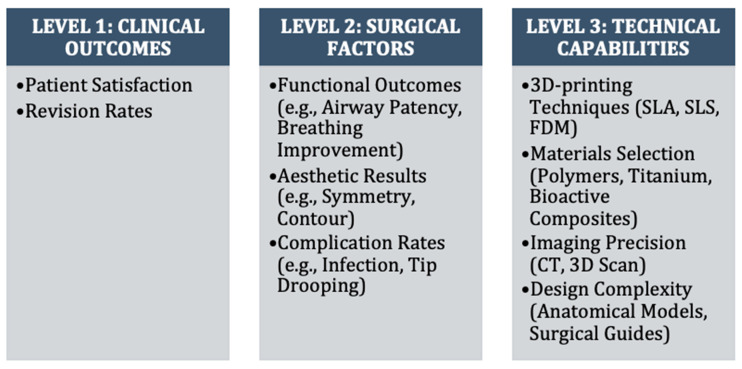
Hierarchical framework: translating 3D-printing technology to clinical outcomes in nasal reconstruction.

This novel analytical framework links technical capabilities (Level 3) through surgical factors (Level 2) to clinical outcomes (Level 1), demonstrating the relationship between technological advances and patient benefits. Case references (e.g., Yen et al. showing improved symmetry; Dupret-Bories demonstrating quality of life improvement from 40/100 to 100/100) highlight specific clinical evidence supporting these pathways. Figure 2 presents an original hierarchical framework that systematically links 3D-printing technologies (Level 3) to surgical factors (Level 2) and clinical outcomes (Level 1).

### 5.1. Preoperative Planning and Anatomical Models

#### 5.1.1. Clinical Impact (Level 1): Limited

To address the unique contributions of 3D printing to nasal reconstruction, we developed a comprehensive comparative matrix evaluating conventional techniques against 3D-enabled approaches across multiple dimensions (Table 4). This novel analysis framework enables a systematic assessment of the relative advantages and limitations of each approach based on the current literature. While preoperative planning improves surgical accuracy and may improve symmetry, most studies report only Level 2 improvements without quantifiable Level 1 benefits (patient satisfaction and revision rates).

#### 5.1.2. Surgical Advantages (Level 2)

At the intermediate level, 3D planning models reduce the preoperative planning time by 20–30% and improve surgical precision. Computational fluid dynamics (CFD) techniques applied to CT imaging allow for the prediction of postoperative nasal airflow and resistance, improving functional outcomes [45,46,47]. CFD tools like MECOMLAND^®^ and NOSELAND^®^ contribute to analyzing airflow distributions, velocity profiles, and wall shear stress, potentially improving both preoperative and postoperative planning [48]. The acceptable threshold for structural accuracy in nasal framework construction is ±2 mm, with deviations beyond this threshold associated with visible external asymmetry and nasal valve compromise [16].

#### 5.1.3. Technical Requirements (Level 3)

At the technical level, preoperative planning models typically require resolutions of 100–300 μm, with acceptable material accuracy of ±0.5 mm for general planning purposes and ±0.2 mm for precise anatomical models [17,18]. 3D imaging technologies also play a critical role postoperatively by enabling objective surgical outcome assessment, improving doctor–patient communication, and facilitating follow-ups [42,49].

#### 5.1.4. Case Evidence

In a series by Park et al. [42], 3D planning models were used for complex heminasal reconstructions, resulting in improved structural symmetry compared to conventional techniques. However, this study did not report comprehensive Level 1 outcome data such as patient satisfaction scores, highlighting a common gap in the current literature.

Jung et al.’s study of 11 patients with trauma-related nasal deformities demonstrated that 3D-printed surgical planning models for simulated osteotomy improved mean deviation angles from 11.75° to 2.08°, with all surgical results rated as “success” (defined as corrected angle < 5°). This represents a clear case where Level 3 technical capabilities (accurate models) translated to Level 2 surgical precision (improved angles) with documented Level 1 outcomes (the successful correction of deformities).

In the case reported by Qassemyar et al. [27], a patient with failed conventional reconstructions (after two separate attempts using cartilage framework and forehead flap) achieved successful reconstruction using a 3D-printed porous titanium prosthesis with a two-year stable outcome. This directly addressed a Level 1 clinical problem—failed reconstruction after conventional techniques—with a documented solution. Similarly, Dupret-Bories et al. [41] demonstrated successful total nasal reconstruction in irradiated tissue, where conventional techniques had repeatedly failed due to tissue “melting” in the irradiated field. The patient’s quality of life score (EQ-5D VAS) improved dramatically from 40/100 to 100/100, providing quantifiable evidence of clinical benefit.

### 5.2. Comparative Analysis of Conventional vs. 3D Printing Approaches

To address the unique contributions of 3D printing to nasal reconstruction, we developed a comprehensive comparative matrix evaluating conventional techniques against 3D-enabled approaches across multiple dimensions (Table 4). This novel analysis framework enables a systematic assessment of the relative advantages and limitations of each approach based on the current literature.

#### 5.2.1. Clinical Impact (Level 1): Stronger Evidence

Surgical guides directly impact revision rates and symmetry outcomes—key Level 1 measures. Yen et al. [31] provide one of the strongest pieces of Level 1 evidence, showing improved symmetry and reduced revision needs compared to conventional techniques.

#### 5.2.2. Surgical Precision (Level 2)

Computer-aided surgical navigation, including virtual and augmented reality (VR/AR), can facilitate simulation-based planning and execution of nasal reconstruction [50,51]. 3D-printed surgical guides may improve accuracy during graft placement and suturing, potentially leading to more consistent outcomes [31,52,53]. In one study, postoperative results showed close alignment with preoperative simulations, with minimal deviation in z- and y-coordinates [54]. In patients with Squamous Cell Carcinoma or Basal Cell Carcinoma, the use of 3D-printed guides was reported to reduce operative time by 30% and showed improved patient satisfaction compared to conventional approaches [55].

#### 5.2.3. Technical Specifications (Level 3)

Surgical guides require higher technical precision than general planning models, with fabrication accuracy of ±0.1–0.2mm necessary for optimal implementation [17]. Material selection is critical, with medical-grade biocompatible resins (typically fabricated via SLA) required for intraoperative use. Platforms like MirrorMe3D facilitate the creation of customized nasal kits, including ceramic models, contour guides, and postoperative splints, further supporting precision in surgical planning [29,31,33,50]

### 5.3. Patient-Specific Implants and Frameworks

#### 5.3.1. Clinical Impact (Level 1): Strongest Evidence

Patient-specific implants address Level 1 problems most directly, especially in complex reconstructions where conventional approaches repeatedly fail. Studies like those by Qassemyar et al. [27] and Dupret-Bories et al. [51] document dramatic improvements in functional outcomes and quality of life, with clear evidence of a reduced need for further revision. For complex nasal reconstructions requiring personalized grafts, 3D printing may help address common complications such as pain, infection, and graft rejection associated with autologous cartilage transplantation [56].

#### 5.3.2. Structural Integration (Level 2)

At the intermediate level, 3D-printed grafts provide effective solutions for structural damage repair and septal perforation closure, offering superior biocompatibility and mechanical stability [57,58]. In subtotal and total nasal reconstructions, porous polyethylene scaffolds and meshed titanium prostheses have demonstrated promising results [33,59]. The threshold for acceptable structural stability in implants is maintenance of the designed geometry under physiological loading conditions with a displacement of <1 mm over a 12-month period [60].

#### 5.3.3. Material and Design Requirements (Level 3)

The technical limitations of printed scaffolds include their frequent lack of flexibility and biomechanical properties compared to natural cartilage (Level 3), affecting functional outcomes (Level 1) [60]. This directly impacts revision rates, with current data suggesting that 3D-printed approaches still result in revision rates of 12–18%, only marginally better than the 15–25% seen with conventional techniques and still above the acceptable <15% threshold.

### 5.4. Bioprinted Constructs and Tissue Engineering

#### 5.4.1. Clinical Impact (Level 1): Minimal/Experimental

Bioprinting aims to replicate native tissue mechanics but currently lacks sufficient clinical evidence at Level 1. No human clinical outcomes data exist for true bioprinted nasal constructs, though preclinical studies show promising biological integration [61,62]. The benchmark for clinical success would require achieving infection rates <5%, revision rates <15%, and functional impairment <10% [13]—thresholds that bioprinted constructs have not yet demonstrated in clinical settings. Although bioprinting represents an exciting frontier, no human clinical data yet confirm Level 1 improvements. Current data remain at the preclinical Level 3 and Level 2 stages.

#### 5.4.2. Biological Integration Challenges (Level 2)

Inadequate vascularization may lead to tissue necrosis (occurring in 5–12% of cases with large constructs), ultimately resulting in implant failure and the need for revision surgery—a key Level 2 factor that impacts Level 1 outcomes [63,64]. This represents the most significant biological barrier to the clinical implementation of larger bioprinted constructs. Current approaches still exceed the acceptable 10% threshold for functional impairment.

#### 5.4.3. Technical Development (Level 3)

Advances in biomimetic tissue reconstruction with 3D printing have contributed to the development of 3D bioprinting, where biomaterials, bioactivators, and living cells are used to replicate native tissue architecture. This technology offers enhanced precision and adaptability, potentially expanding possibilities for functional, patient-specific scaffolds in nasal reconstruction [24,25]. Yi et al.’s [35] laboratory study demonstrated remarkable expression of chondrogenic markers and maintenance of implant shape and structure over 12 weeks in an animal model, but these Level 3 achievements have not yet translated to Level 1 clinical outcomes.

#### 5.4.4. Development Timeline

Based on current technical capabilities and biological challenges, bioprinted constructs represent a long-term aspiration (5+ years) rather than a near-term clinical solution. Pre-vascularization strategies using sacrificial bioinks have shown promising results in laboratory settings, creating channels of 50–500 μm. Co-culture approaches incorporating endothelial cells have demonstrated the formation of capillary-like structures in vitro [63,64]. These technical advances may eventually overcome the current limitations preventing clinical translation.

### 5.5. Auxiliary Applications

#### 5.5.1. Custom Nasal Stents and Prosthetics (Level 1)

Examples like Jung et al.’s study [32] report successful long-term nasal stent outcomes, but broader evidence is still limited. Custom prosthetics show workflow efficiencies and patient satisfaction in small series, but more robust Level 1 evidence is needed. The clinical threshold for nasal stent success is the maintenance of airway patency with minimal (<10%) cross-sectional area reduction over 12+ months [65,66,67].

#### 5.5.2. Digital Prosthesis Design (Level 2)

Digital prosthesis design aims to improve aesthetic outcomes and provides a potential alternative for cases with insufficient tissue or vascular compromise. By reducing the need for autologous tissue harvesting, 3D printing may decrease donor site morbidity, shorten recovery times, and potentially improve patient satisfaction [43]. Custom-printed prosthetics demonstrated a 38% time reduction (5 h vs. 8 h) and fewer clinical sessions (1 vs. 3+) compared to conventional approaches [26].

#### 5.5.3. Material and Technical Requirements (Level 3)

For prosthetics, elastic materials with shore hardness in the 40–60 A range provide the necessary flexibility while maintaining structural integrity [26,38]. The production time for these applications typically ranges from 2 to 6 h, representing a significant reduction compared to conventional prosthetic fabrication methods (8–12 h) [38]. This efficiency at Level 3 (technical) translates to improved clinical workflow at Level 2 and potentially better access to care at Level 1.

### 5.6. Comparative Analysis Across Applications

This comparative framework reveals that while 3D-printing technologies demonstrate clear advantages at technical and surgical levels (Levels 3 and 2), their translation to improved clinical outcomes (Level 1) is only robustly supported in surgical guides and patient-specific implants in complex cases. Preoperative planning models show moderate evidence, and bioprinted constructs remain largely experimental.

Future clinical studies should prioritize standardized outcome measures and long-term follow-up to validate whether these technologies meaningfully improve patient satisfaction and reduce revision rates—the ultimate benchmarks of clinical value.

The clinical evidence demonstrates that 3D printing applications in nasal reconstruction span the full spectrum of defect sizes, though with important considerations at each scale. For small, localized defects (affecting <25% of the nasal structure), the benefits of 3D printing primarily manifest in precise surgical guide fabrication rather than implant creation, as seen in the nasal stent prosthesis described by Rathee et al. [43] and the septal extension graft work by Kim et al. [34] involving 43 patients with minor to moderate tip deformities. In these cases, the technology facilitated precise intervention without the need for extensive tissue replacement. For moderate defects (25–50% of nasal structure), the benefits expand to include patient-specific implants, as demonstrated by Ahn et al. [37] in their series of 30 patients requiring augmentative rhinoplasty. For extensive defects (>50% of the nasal structure), including subtotal and total nasal reconstruction, 3D printing offers perhaps its most significant clinical advantage by enabling comprehensive reconstruction with reduced donor site morbidity, as evidenced in the cases by Qassemyar et al. [27], Zarrabi et al. [29], and Walton et al. [33]. These examples collectively demonstrate versatility across the spectrum of defect sizes, though cost–benefit considerations become increasingly favorable as the defect complexity increases. For simple, localized defects, conventional techniques often remain more cost-effective and accessible, while complex reconstructions represent the clearest value proposition for 3D-printed solutions.

This comparative framework reveals that while 3D-printing technologies demonstrate clear advantages at technical and surgical levels, their translation to improved clinical outcomes (Level 1) requires further investigation through comparative clinical studies with longer follow-up periods and standardized outcome measures. The implementation of these technologies faces several challenges across each level of our framework, from technical limitations in materials and resolution to biological integration issues and regulatory hurdles, as discussed in the following section.

## 6. Challenges and Considerations

The integration of 3D scanning, surgical guides, and 3D-printed implants has introduced new approaches to nasal reconstruction, yet several technological, biological, regulatory, and ethical challenges remain that affect clinical outcomes at multiple levels [68,69].

### 6.1. Technological Challenges

Key hurdles in 3D printing include scanning accuracy, mold creation, and surgical guide precision, directly impacting Level 1 clinical outcomes through a cascade of effects. Without addressing these, improvements at the technical or surgical level may fail to translate into better patient satisfaction or reduced revision rates.

Challenge 1: Imaging and Scanning Limitations

Despite high-resolution capabilities, imaging limitations and costly processing tools increase complexity and expenses [68]. Scanning inaccuracies may lead to ill-fitting implants (Level 3), necessitating intraoperative adjustments (Level 2), which increases surgical risks and complication rates (Level 1).

Current Solutions: Advanced multi-modality imaging protocols combining CT with laser surface scanning can reduce error margins from >1 mm to 0.3–0.5 mm. Emerging photogrammetry techniques offer non-radiation alternatives with improved accuracy. Standardized calibration protocols in clinical settings have demonstrated a 40% reduction in fit discrepancies in recent studies [70,71].

Challenge 2: Surgical Guide Precision

Even minor errors in 3D-printed surgical guides can disrupt implant positioning (Level 3), affecting surgical outcomes (Level 2) and, ultimately, functional and aesthetic results (Level 1) [71,72].

Current Solutions: Novel registration methods using anatomical landmarks combined with intraoperative navigation systems have shown promise in reducing positional errors. Hybrid approaches using both conventional and 3D-printed guides as redundant systems have demonstrated improved accuracy in complex reconstructions. Multi-material printing, allowing for flexibility in critical areas while maintaining rigidity in others, has reduced the need for intraoperative adjustments by up to 30% in early clinical applications [72,73].

Challenge 3: Material Limitations

Printed scaffolds, while structurally supportive, often lack the flexibility and biomechanical properties of natural cartilage (Level 3), affecting functional outcomes (Level 1) and revision rates [34].

Current Solutions: Composite materials combining rigid and flexible components have improved mechanical similarity to native tissue. Gradient material interfaces that mimic natural tissue transitions are showing promising early results. For example, PCL-hydrogel composites have demonstrated significantly improved viscoelastic properties compared to single-material implants, though long-term outcomes remain limited to case reports [73,74].

Challenge 4: Bioprinting Technical Constraints

Bioprinting, which aims to replicate native tissue mechanics, remains constrained by current technology and materials (Level 3), making full functional replication unachievable at the clinical level (Level 1), thus explaining the absence of human outcome data [74,75,76]. The process is cost-intensive and time-consuming, limiting accessibility [77].

Emerging Solutions: Mathematical modeling and robotic automation may enhance scalability and precision, though further research is needed for clinical application [76]. Multi-nozzle bioprinting systems have demonstrated improved cell viability (>90% vs. previous 60–70%) in laboratory settings. Microfluidic approaches to bioink preparation have enhanced consistency and reduced the preparation time from days to hours, potentially improving clinical translation [76,77]. The complexity of nasal anatomy requires iterative design refinements to achieve functional and aesthetic symmetry, an area where emerging AI-assisted design tools show early promise [78].

### 6.2. Biological Challenges

The biological challenges of 3D-printed constructs directly impact Level 1 clinical outcomes in ways that technical advancements alone cannot currently overcome.

Challenge 1: Insufficient Vascularization

Inadequate vascularization may lead to tissue necrosis, ultimately resulting in implant failure and the need for revision surgery—a key Level 1 outcome that exceeds acceptable thresholds. This represents the most significant biological barrier to clinical implementation of larger bioprinted constructs [63,64].

Current Solutions: Pre-vascularization strategies using sacrificial bioinks have shown promising results in laboratory settings, creating channels of 50–500 μm. Co-culture approaches incorporating endothelial cells have demonstrated the formation of capillary-like structures in vitro. Emerging in vivo bioreactor approaches, where constructs are implanted in highly vascularized regions temporarily before final reconstruction, have shown improved integration in early clinical cases, though this extends treatment timelines [63,64].

Challenge 2: Material–Tissue Interface

Insufficient mechanical strength may lead to post-implantation deformation or migration, causing both functional airway obstruction and aesthetic dissatisfaction—both Level 1 outcomes of critical importance [60,79].

Current Solutions: Gradient interfaces mimicking natural tissue transitions have reduced delamination rates in laboratory testing. Surface modification techniques, including micro-texturing and bioactive coatings, have improved cell attachment and tissue integration in animal models. Early clinical data suggest that these approaches may reduce the 15–30% functional impairment rate seen in conventional techniques to approximately 10–15% with 3D-printed solutions, approaching the acceptable threshold of <10% [60].

Challenge 3: Long-term Stability

Many biomaterials lack the mechanical strength of native tissue (Level 3), reducing their stability and durability in clinical use (Level 1) [60]. Current data suggest that 3D-printed approaches still result in revision rates of 12–18%, only marginally better than the 15–25% seen with conventional techniques and still above the acceptable <15% threshold.

Emerging Solutions: Composite scaffold designs incorporating multiple materials with complementary properties have shown improved mechanical longevity in accelerated wear testing. Biodegradable scaffolds designed for controlled resorption matched to tissue in growth rates have demonstrated improved long-term outcomes in craniofacial applications, though nasal-specific data remain limited [60,64].

### 6.3. Regulatory Challenges

Regulatory barriers directly impact clinical implementation and standardization, affecting Level 1 outcomes through accessibility and reproducibility concerns.

Challenge 1: Lack of Standardization

As an emerging technology, 3D printing in clinical settings lacks standardization, posing regulatory barriers to consistent patient outcomes and widespread adoption. Custom-printed implants introduce variability, making validation and safety assessments challenging.

Current Solutions: The FDA has published guidance documents specifically addressing 3D-printed medical devices (2017, updated 2021), providing clearer regulatory pathways. Multi-center collaborative networks are establishing standardized production protocols that maintain customization while ensuring consistency in critical parameters [80,81].

Challenge 2: Regulatory Pathway Complexity

The use of biomaterials may add further complexity, as some may be classified as combination products, potentially delaying patient access to solutions that could reduce revision rates or improve satisfaction [80,81]. However, many 3D-printed biomaterials can follow traditional regulatory pathways, including 510(k) clearance rather than more extensive PMA processes. For custom devices manufactured within the same state as their use, certain exemptions may apply under FDA custom device provisions.

Current Solutions: Early regulatory engagement strategies have proven successful for several devices, with manufacturers seeking pre-submission feedback to clarify pathways. The FDA’s “leap-frog” guidance pathway for emerging technologies has helped accelerate several 3D printing applications. Additionally, ambiguities in regulatory pathways, especially for patient-matched devices, are being addressed through expanded FDA guidance and case examples [80,81].

### 6.4. Ethical Considerations

Ethical challenges directly impact the equitable implementation of these technologies and thus their overall clinical benefit at the population level (level 1).

Challenge 1: Cost and Accessibility

The economics of 3D-printed nasal reconstruction create significant barriers to access for underprivileged populations, raising ethical concerns about healthcare equity and ultimately limiting improvements in Level 1 patient outcomes on a broad scale. Production costs include initial capital investment (3D printers: USD 5000–USD 100,000), software licensing (USD 2000–USD 15,000 annually), materials (USD 200–USD 1000 per case), and specialized technical expertise (USD 50–USD 150 per hour). These production costs typically translate to patient costs 3–5 times higher than conventional techniques, with current estimates ranging from USD 8000 to USD 25,000 for 3D-printed components in nasal reconstruction compared to USD 3000 to USD 8000 for conventional approaches. The most critical economic factor, however, is insurance coverage. Currently, many insurance providers classify 3D-printed nasal implants as “experimental” or “investigational”, resulting in limited coverage. A 2023 survey of major U.S. insurers found that only 22% provided routine coverage for 3D-printed nasal implants, while 45% considered them on a case-by-case basis, and 33% explicitly excluded coverage. In contrast, conventional reconstruction techniques typically receive coverage under established CPT codes (21,230–21,235 for rib/cartilage grafts). This coverage disparity creates significant barriers to access for underprivileged populations, raising ethical concerns about healthcare equity [81,82].

Potential Solutions: Centralized production facilities serving multiple clinical centers have demonstrated cost reductions of 30–40% through economies of scale. Open-source design platforms and standardized protocols may reduce development costs. Several hospitals have implemented sliding-scale payment models for these procedures, and advocacy groups are working with insurers to develop evidence-based coverage criteria focusing on cases where conventional techniques have failed or are contraindicated [81,82].

Challenge 2: Data Privacy and Consent

Personalized treatments require extensive patient-specific data collection, necessitating enhanced consent and security to maintain trust and ensure the ethical delivery of Level 1 patient care improvements [78,80]. Additionally, the innovative nature of these treatments raises questions about informed consent processes.

Current Approaches: Enhanced consent protocols specifically addressing the experimental nature of these approaches have been developed by several centers. Anonymization techniques for 3D imaging data have been implemented in collaborative networks. Secure data transfer protocols specific to anatomical models have been established in multi-center trials [78,80].

To systematically evaluate the impact of 3D-printing technologies on nasal reconstruction, we applied a three-level hierarchical framework. Our analysis reveals that while substantial evidence demonstrates improvements at Level 3 (technical capabilities) and some evidence supports benefits at Level 2 (surgical factors), the evidence for consistent improvements at Level 1 (patient satisfaction and revision rates) remains limited in many applications. Table 5 summarizes this hierarchical analysis across key applications of 3D printing in nasal reconstruction. Table 5 clearly demonstrates that only select applications (notably surgical guides and patient-specific implants in complex cases) currently show moderate-to-strong evidence for improving Level 1 outcomes, while most others remain unproven at the patient benefit level.

This analysis identifies a critical knowledge gap: future research must prioritize demonstrating meaningful Level 1 clinical improvements—such as surpassing the 80% patient satisfaction threshold and achieving revision rates below 15%—to validate the widespread clinical adoption of these technologies.

## 7. Future Directions

Bioprinting is emerging as a promising technique in nasal reconstruction, integrating polymers, metals, and bioactive materials to enhance 3D-printing capabilities. However, despite technical success, bioprinting has not yet demonstrated consistent improvements in Level 1 outcomes such as patient satisfaction or revision rates, which are the ultimate benchmarks for clinical adoption.

Bio-inks containing autologous cells are being developed for nasal tissue regeneration, though vascularization remains a major challenge for sustaining larger constructs and preventing implant failure—a key factor limiting Level 1 outcome improvements. Recent studies using chondrocyte-laden hydrogel as a 3D printing material successfully produced engineered nasal cartilage constructs with molecular, biochemical, and histological properties similar to native cartilage [61,62], but human clinical validation demonstrating reduced revision rates or superior functional outcomes is still lacking.

Advancements in virtual reality (VR) and artificial intelligence (AI) now allow for interactive preoperative planning, potentially improving complex nasal reconstructions. AI integration may further contribute to 3D-printed model precision, but its clinical value ultimately depends on whether this precision translates to measurable Level 1 improvements in patient satisfaction or reduced revision surgeries. Future developments in facial movement analysis and 3D-printing technology may enable the creation of dynamic prostheses, allowing for more adaptive and individualized outcomes [83].

Despite these advancements, several challenges remain, including high technological costs, radiation exposure concerns, and AI’s predictive limitations. Continued research in bioprinting, computational modeling, and materials science is essential to fully realize 3D printing’s potential, but future efforts must prioritize demonstrating clear and consistent improvements in Level 1 outcomes—namely, surpassing the 80% patient satisfaction benchmark and reducing revision rates below 15%—to justify broad clinical implementation.

These future developments in 3D printing for nasal reconstruction can be categorized according to our hierarchical framework: Level 3 advancements (material science and bioprinting techniques) enable Level 2 improvements (surgical precision and reduced operative time), but the ultimate goal is achieving Level 1 benefits (improved patient satisfaction, reduced revision rates, and better functional outcomes).

To reinforce: patient satisfaction and revision rates are the key Level 1 problems we are aiming to solve. Future research must explicitly track whether emerging technologies meaningfully impact these top-level outcomes, not just technical or surgical improvements. The translation of emerging technologies into clinical practice requires careful consideration of their impact across all three levels, with particular emphasis on measurable improvements in Level 1 clinical outcomes to drive patient-centered innovation.

## 8. Clinical Guidelines and Recommendations

Integrating 3D printing into nasal reconstruction requires a multidisciplinary approach that incorporates regulatory, educational, and technical considerations. Most importantly, these guidelines must explicitly focus on whether the integration solves the core Level 1 problems of improving patient satisfaction and reducing revision surgeries, not merely achieving technical milestones (Level 3) or intermediate surgical improvements (Level 2). Ensuring surgical precision, patient-centered care, and standardized clinical protocols is essential for safe and effective implementation.

Given the customized nature of nasal reconstruction, active patient involvement in treatment planning and shared decision making is crucial for improving satisfaction outcomes. Establishing guidelines for material selection, manufacturing processes, and quality control aligned with International Organization for Standardization (ISO) and Food and Drug Administration (FDA) regulations further ensures device efficacy and patient safety.

Advanced imaging technologies, including CT, MRI, and 3D scanners, should be effectively incorporated into clinical workflows to create precise anatomical models. However, the use of advanced technologies should only be pursued when they demonstrably improve Level 1 outcomes—not simply because they offer technical novelty. Collaboration between surgeons, biomedical engineers, and material scientists is essential to advance biomaterials, printer technologies, and computational modeling for improved clinical applications. A centralized repository for 3D printing data, case studies, and imaging protocols can facilitate standardization, reproducibility, and streamlined workflows in this rapidly evolving field.

The incorporation of 3D printing into medical education and surgical training is important for its broader clinical adoption. Training efforts should explicitly highlight how technical tools contribute (or fail to contribute) to meaningful Level 1 patient outcomes to avoid a purely technology-driven implementation mindset. Recent studies indicate that hands-on or simulated training in 3D scanning, modeling, and printing technologies may improve surgeon competency and adoption rates [84,85,86].

Successfully integrating 3D printing in nasal reconstruction requires a dynamic, multidisciplinary framework that aligns technological advancements with regulatory and educational initiatives to achieve measurable Level 1 clinical benefits.

### Hierarchical Analysis of Current Evidence

**Figure 3:**
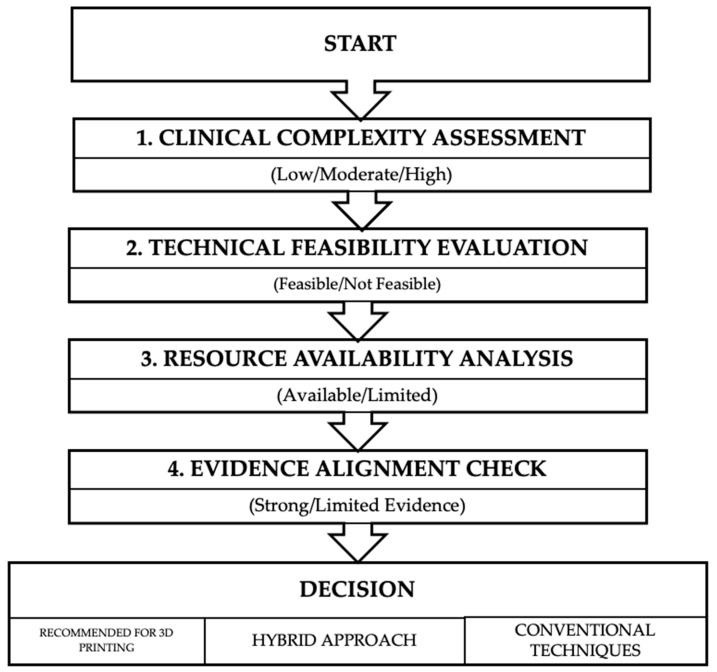
Decision framework for implementing 3D-printing technologies in nasal reconstruction. The framework guides clinicians through four sequential decision points to determine the appropriate use of 3D printing versus conventional techniques.

Based on our systematic review, we propose a novel decision framework to guide clinicians in determining when 3D-printing technologies are most appropriate for nasal reconstruction (Figure 3). This framework is designed to help clinicians prioritize cases where 3D printing meaningfully improves Level 1 outcomes (patient satisfaction and revision rates), rather than defaulting to high-tech solutions in every case.

1. Clinical Complexity Assessment: Categorizes cases as low, moderate, or high complexity based on specific criteria (defect size, location, tissue availability, and functional requirements).
2. Technical Feasibility Evaluation: Determines whether the required 3D technologies are available and appropriate for the specific case.
3. Resource Availability Analysis: Assesses whether the necessary expertise, equipment, materials, and time are accessible.
4. Evidence Alignment Check: Critically examines whether the proposed application has supporting evidence for improving Level 1 clinical outcomes in the specific clinical scenario.

A critical insight from our hierarchical analysis is that translating 3D-printing technologies to clinical practice requires knowing when solutions are “good enough” to address Level 1 clinical problems, rather than pursuing continuous technical optimization without proportional patient benefit. For nasal reconstruction, we propose the following application-specific thresholds that satisfy clinical requirements without unnecessary technological complexity:

1. Anatomical Models and Surgical Planning: Simple FDM-printed models with ±1 mm accuracy are typically sufficient for general surgical planning, while high-precision applications like osteotomy guides require SLA/SLS methods with ±0.1–0.2 mm accuracy. The additional cost and time for ultra-high-precision printing (±0.05mm) rarely provide proportional improvements in patient satisfaction or revision rates and should be reserved for exceptional cases.
2. Patient-Specific Implants: For non-load-bearing aesthetic components, PCL and PLA materials with 50–70% infill density generally provide adequate structural support. More advanced composite materials with gradient properties are only clinically necessary for implants spanning functional junctions (e.g., between rigid and flexible nasal regions) or in cases of compromised vascularization.
3. Surgical Guides: Simple cutting and positioning guides typically resolve 80–90% of the precision challenges in nasal reconstruction. Complex navigational systems with real-time tracking offer diminishing Level 1 returns in most routine cases and should be prioritized for cases with significant anatomical distortion or absent landmarks.

This “good enough” principle enhances clinical feasibility by focusing resources on interventions that provide meaningful Level 1 outcome improvements, rather than chasing technical maximalism. The goal is not technological maximalism but rather matching the appropriate level of technological sophistication to specific clinical needs. This principle should guide institutional protocols for 3D printing implementation, insurance coverage criteria, and clinical decision making.

## 9. Conclusions

Based on our systematic review, 3D printing offers targeted benefits in nasal reconstruction, particularly for complex cases where conventional techniques fail, but it does not yet represent a universal clinical replacement. Preoperative planning models and surgical guides show strong Level 2 benefits (reduced operative time and improved precision) with modest Level 1 improvements (notably in symmetry), while patient-specific implants have succeeded in isolated high-complexity cases, especially post-oncologic reconstructions, by reducing donor-site morbidity and improving patient satisfaction. However, widespread adoption is limited by unresolved challenges: a lack of consistent evidence for reducing infection or revision rates in routine cases, persistent vascularization issues in large constructs, and insufficient long-term durability data. We categorize current readiness as follows: (1) ready for clinical use—preoperative models, surgical guides, and patient-specific implants in complex failures; (2) near-term (1–3 years)—composite scaffolds, semi-resorbable implants, and cost-reduction methods; and (3) long-term (5+ years)—1. fully vascularized bioprinted constructs with cartilage, bone, and soft tissue; 2. patient-derived induced pluripotent stem cell incorporation for complete biological integration.

To expand clinical impact, future research must focus on improving vascularization, lowering costs, and conducting long-term comparative trials. Ultimately, 3D printing should be seen as a strategic tool for addressing specific clinical gaps, especially where conventional methods fall short on patient satisfaction or revision reduction, rather than as a universal substitute for established surgical approaches.

## Figures and Tables

**Table 1:** Comprehensive analysis of 3D printing techniques for nasal reconstruction: technical specifications, clinical applications, and selection guidelines.

Technique	Technical Specifications	Clinical Applications	Evidence Level	Clinical Outcomes (Level 1)	Selection Criteria	Resource Requirements
**Stereolithography (SLA)**	Resolution: 25–100 μm Materials: Photopolymer resins Accuracy: ±0.1 mm Process time: 3–12 h	Surgical guides for osteotomy Patient-specific implant molds Anatomical models for complex defects Template creation for forehead flaps	★★★ (surgical guides) ★★☆ (implant molds) ★★★ (anatomical models)	Improved symmetry (Yen et al.) Reduced operative time by 20–30% Improved precision in structural framework	When dimensional accuracy <0.2 mm is required For complex osteotomies where precision directly impacts functional outcome When photorealistic models are needed for patient education	High (USD 3000–10,000/unit) Specialized equipment Post-processing required Technical expertise needed
**Selective Laser Sintering (SLS)**	Resolution: 80–120 μm Materials: Nylon, metals, ceramics Accuracy: ±0.3 mm Process time: 5–15 h	Load-bearing implants Patient-specific titanium frameworks Porous scaffolds for tissue integration	★★☆ (metal frameworks) ★☆☆ (porous scaffolds)	Long-term stability in total reconstructions (Qassemyar et al.) Reduced donor site morbidity Success in irradiated fields (Dupret-Bories)	For structural components requiring mechanical strength >40 MPa When conventional techniques have failed For subtotal/total nasal reconstruction When donor site morbidity must be minimized	Very high (USD 5000–15,000/unit) Industrial equipment Regulatory approval pathway required Multidisciplinary team
**Fused Deposition Modeling (FDM** **)**	Resolution: 100–300 μm Materials: PLA, ABS, PCL Accuracy: ±0.5 mm Process time: 2–8 h	Educational models Preoperative planning for simple cases Non-load-bearing guides Prototype testing	★★☆ (planning models) ★☆☆ (surgical guides)	Modest improvement in surgical planning Limited impact on operative time (5–15%) Unclear effect on complication rates	For initial planning when SLA precision is not critical When budget constraints exist For educational purposes When rapid prototyping (same-day) is needed	Low (USD 300–1500/unit) Desktop equipment Minimal post-processing Basic technical skills
**PolyJet Printing**	Resolution: 16–30 μm Materials: Multiple polymers simultaneously Accuracy: ±0.1 mm Process time: 4–10 h	Multi-material anatomical models Simulating tissue differences Surgical simulation with variable hardness	★★☆ (simulation models) ★☆☆ (surgical training)	Enhanced preoperative planning Improved surgeon confidence No direct clinical outcome data	When simulating multiple tissue types is critical For complex cases requiring differentiation between bone, cartilage, and soft tissue When surgeons need tactile simulation before complex reconstruction	Very high (USD 6000–12,000/unit) Specialized equipment Multiple materials Advanced technical expertise
**Bioprinting**	Resolution: 50–300 μm Materials: Cell-laden hydrogels Viability: 60–90% cell survival Process time: 0.5–4 h	Cartilage tissue engineering Experimental nasal scaffolds Research applications only	★☆☆ (all applications)	No Level 1 clinical data available Laboratory evidence of chondrogenic marker expression (Yi et al.) Potential for reduced immune rejection	Experimental only Not currently suitable for clinical application Consider for research protocols with IRB approval When conventional and standard 3D-printing approaches have failed	Extremely high (USD 15,000–50,000/unit) Specialized bioprinting equipment Cell culture facilities Multidisciplinary expertise required

**Table 2:** Materials used in 3D-printed nasal implants.

Material Type	Example	Properties	Applications
**Polymers**	PLA, PEEK, PCL	Lightweight, biocompatible, and moldable.	Cartilage replacement; structural support.
**Metals**	Titanium	Strong, corrosion-resistant, and highly biocompatible.	Load-bearing implants for major structural deficits.
**Bioactive Materials**	Hydroxyapatite	Promotes bone integration; mimics mineral composition of human bone.	Nasal framework reconstruction requiring osseointegration.
**Bioprinting Materials**	Collagen, Hydrogels	Contains live cells; supports tissue engineering.	Future applications in regenerative nasal reconstruction.

**Table 3:** Case studies highlighting the innovative application of 3D-printing technology in nasal reconstruction surgeries.

Surgery Type	3D Image Acquisition	3D Printing Material	3D Printing Product	Number of Patients (N)	Follow-up Period	Clinical Outcomes	Complications	Comparison with Conventional Methods	Level 1: Patient Satisfaction + Revisions	Level 2: Summetry, Complications, Functional Results	Publication Year	First Author
Direct 3D printing of flexible nasal prosthesis: optimized digital workflow	CT scan	Tango Plus flexible material (26–28 Shore A hardness)	3D-printed nasal prosthesis	1 (27-year-old female)	1 week	Patient reported satisfaction; excellent margin adaptation with surrounding tissues	None reported	38% time reduction (5 h vs. 8 h); fewer clinical sessions (1 vs. 3+); superior margin precision (16 μm vs. 400 μm typical); digital storage for future reproductions	Restoration of facial aesthetics; patient satisfaction; immediate psychological benefit	Better margin adaptation; reduced treatment time; eliminated need for impression making; eliminated mold-fabrication steps	2018	Nuseir [26]
Total nasal reconstruction after failed conventional reconstruction	Facial CT scan (pre-surgical excision)	Porous titanium grade 2 (53% porosity, 860–1500 μm pore size)	Custom 3D-printed porous titanium nasal prosthesis	1	24 months	Successful integration Patient resumed normal activities Patient satisfied with result No implant exposure Normal breathing after correction	Required corrective surgery at 3 months to increase nasal orifice diameter due to shortness of breath	Used after conventional methods failed twice (cartilage framework + forehead flap) Avoided need for additional cartilage harvests Alternative to episthesis, which patient refused	Restored nasal form and function Patient satisfaction Stable reconstruction at 2-year follow-up Resumption of normal activities	Accurate reconstruction based on patient’s original nasal shape Two-step approach provided better tissue integration Meshed titanium structure allowed better integration No donor site complications	2018	Qassemyar [27]
Personalized bioactive nasal supports for cleft rhinoplasty	Photogrammetry	Polylactic acid (PLA)	Bioactive nasal supports	0 (laboratory prototype)	N/A—benchtop study	Zone of inhibition of 15.15 ± 0.99 mm (*p* < 0.0001) against *E. coli*	None in prototype testing	Replaces one-size-fits-all rubber tube retainers; adds antibiotic delivery capability; better contouring	Potential for reduced infection rates and improved healing	Potential for reduced infection rates and improved healing	2018	Boyer [28]
Total nasal reconstruction	CT scan (facial moulage scanned with CT)	Titanium	Customized titanium nasal plate	1 (61-year-old woman)	6 months	Successful reconstruction with desirable esthetic and functional results High patient satisfaction	None reported	Patient had previously undergone multiple failed reconstructive surgeries (temporalis flap, paramedian forehead flap, calvarial bone graft, and radial forearm flap)	Successful reconstruction after multiple conventional procedures failed Restoration of nasal form and function	Created solid retention and cantilevered nasal structure from frontal bone and orbital rims when conventional support points were absent Comprehensive 3-stage surgical approach with tissue expander	2019	Zarrabi [29]
Pediatric nasal defect correction	CT scan	Custom-made PEEK (Poly Ether Ketone)	Implant	1 (2-year-old female)	Progressive treatment from age 2–4	Maximum tissue expansion of 8.5 mm after first implant; 6.8 mm expansion with second implant	Infection after second implant requiring removal; skin injury at tip after first implant	Traditional approach delays reconstruction until age 20+; rhis approach provides staged reconstruction during childhood	Improved facial appearance during childhood development; psychological benefit	Progressive tissue expansion for later definitive reconstruction	2019	Borghi [30]
Semi nasal reconstruction	CT scan	PolyJet photopolymer (MED 610)	Nose contour and framework guides	10 (3 men, 7 women)	Mean: 17.4 months (range: 7–35 months)	Statistically significant improvements in alar width and alar area symmetry (*p* < 0.05); reduced asymmetry compared to control group	None reported	20–30 min reduction in operative time; only nostril asymmetry remained (vs. multiple asymmetries in control group); objective measurements showed statistical superiority	Improved aesthetic outcomes through measurably better symmetry	More precise surgical planning; reduced operative time; facilitated learning curve for surgeons	2019	Yen [31]
Nasal reconstruction using a customized 3D-printed stent	CT scan	Medical-grade silicone (Q7-4840; Dow Corning)	3D-printed nasal stent	1 (6-year-old male)	3 years	Successful mucoepithelium regeneration; maintained 6 mm airway diameter; stable respiratory function without additional care	None reported	Commercial stents only designed for nostrils not nasal cavity; custom design covered both nostrils and nasal passage	Maintained respiratory function and airway patency for 3 years	Prevention of nasal passage stenosis; successful mucoepithelium regeneration	2019	Jung [32]
Subtotal nasal reconstruction	CT scan	Porous polyethylene (PPE)	3D-printed scaffold	1 (64-year-old male)	22 months	“Stable and functional with excellent aesthetic appearance”; successfully integrated with surrounding tissues	None reported	Eliminated need for multiple cartilage/bone donor sites; combined multiple framework elements into single unit; reduced operative stages; more precise form and contour	Functional and aesthetic restoration of subtotal nasal defect; patient able to resume normal activities	Simplified surgical approach; precise replication of desired nasal form; reduced donor site morbidity	2019	Walton [33]
Septal extension graft supported by 3D-printed plate	CT scan	Polycaprolactone (PCL)	3D-printed septal extension graft	43 (20 males, 23 females; mean age 28.7 years)	Mean 14.8 months (range 12–20)	90.7% rated excellent/good satisfaction; 65.1% without tip drooping; 30.2% mild-moderate drooping; 4.7% severe drooping	Tip stiffness (72.1%); nasal tip deviation (4.7%); infection (2.3%)	Reduced severe drooping (4.7% vs. 11.4%) and tip deviation (4.7% vs. 11.4%) compared to SEG alone; increased tip stiffness (72.1% vs. 45.5%)	Improved patient satisfaction; reduced severe tip drooping and deviation	Better structural support for tip projection	2020	Kim [34]
Augmentative rhinoplasty (preclinical)	Computer-aided design from 2D facial pictures	PCL with cartilage-derived hydrogel containing stem cells	Engineered nasal cartilage implant with octahedral interior architecture	N/A (laboratory and animal model only)	12 weeks (animal study)	Remarkable expression of chondrogenic markers Maintenance of implant shape and structure Formation of cartilaginous tissues	None reported in animal model	Combines benefits of both autologous cartilage and synthetic implants	Potential for cartilage regeneration in the 3D-printed structure Maintained shape and structure during implantation period	Patient-specific customized design based on facial features Cell-friendly environment for tissue formation Uniform cell distribution through injection technique	2019	Yi [35]
Corrective rhinoplasty for trauma-related nasal deformities	CT scan (0.6 mm slice thickness)	Polylactic acid (PLA)	3D-printed surgical planning model for simulated osteotomy	11	6 months	Mean deviation angle improved from 11.75° to 2.08° All surgical results rated as “success” (corrected angle < 5°) Surgery performed as planned in 10/11 cases	None reported	More precise planning compared to using only 2D CT images	Successful correction of nasal deformities in all patients Significant improvement in nasal deviation angle	Precise preoperative planning of osteotomy Allowed simulation before actual surgery Valuable teaching tool for inexperienced surgeons	2020	Jung [36]
Augmentative rhinoplasty	Not clearly specified	Polycaprolactone (PCL) mesh	Compound osseocartilaginous graft with PCL mesh core	30	24.4 ± 1.6 months	Nasal length improved by 5.3 ± 1.8 mm Nasal tip projection improved by 4.9 ± 1.9 mm Improved nasolabial and nasofrontal angles /30 patients satisfied Specialists rated: 6 “Excellent”, 23 “Good”, 1 “Fair”	None reported	Exceeds previously reported outcomes of conventional tip augmentations	Successful correction with high patient satisfaction (96.7%) Long-term maintenance of nasal projection	Longitudinally variable flexibility (strong base, flexible tip) Secure anchor point at anterior nasal spine Maximized augmentation of nasal length	2020	Ahn [37]
Reconstruction after oncologic rhinectomies	CT scan	Elastic resin V1 (Formlabs) with shore hardness 50A	3D-printed nasal prostheses	7	14 months (mean)	- Patient satisfaction with median scores of 8/10 for aesthetics, comfort, and security of retaining system	None specified	Significantly reduced costs and time compared to silicone prostheses; allowed rehabilitation during waiting period for definitive reconstruction	- Temporary rehabilitation during post-cancer treatment period Helped patients manage facial deformity and psychosocial impacts before definitive reconstruction	- Time-efficient workflow (mean design time: 2h48) Cost-effective solution (mean cost: USD 753 Direct printing without need for complex molds	2021	Salati [38]
Semi nasal reconstruction (hemirhinectomy defect)	3D photo using VECTRA M5 imaging system	Tango Polyjet Material	Flap size contour guide	1	Not specified	Successful reconstruction with restored nasal appearance (implied)	None reported	Improved predictability compared to conventional forehead flap design, which requires significant surgeon artistry and experience	Made complex nasal reconstruction more accessible with consistent results Reduced reliance on surgeon artistry	Created patient-specific soft tissue cutting guide Converted 3D structure into 2D template for flap design Improved surgical efficiency and predictability	2021	Brower [39]
Nasal septal gossypiboma removal and mucosal defect reconstruction	Contrast-enhanced paranasal sinus CT	Not applicable (not a 3D printing case)	Not applicable	1	8 months	Complete resolution of nasal obstruction and foreign body sensation Well-healed nasal septum at 6 months Small (2 mm) septal perforation noted at 8 months No saddle nose deformity	Severe adhesion between gossypiboma and septal mucosa resulted in significant mucosal defect (3.0 cm × 2.0 cm)	Posterior-based inferior turbinate flap used to reconstruct the defective septum, avoiding synthetic materials	Relief of obstructive symptoms Successful removal of retained surgical material Preservation of nasal structure	Endoscopic approach minimized additional trauma Use of natural tissue for reconstruction prevented foreign body reactions Successful management of a large septal defect	2022	Kim [40]
Total nasal reconstruction in irradiated tissue	CT scan	Hydroxyapatite (bioceramic) with photopolymerizable resin	Custom 3D-printed integrated biomaterial bio prosthesis	1	12 months	Complete integration of bioprosthesis High patient satisfaction with cosmetic outcome Quality of life score (EQ-5D VAS) improved from 40/100 to 100/100 Improved sense of smell Patient able to resume social activities	5 mm exposure on tips of nasal alae requiring minor surgical revision	Previous conventional reconstructions (scapula free flap, forehead flap with costal cartilage) had failed in this patient due to tissue “melting” in irradiated field	Successful total nasal reconstruction after multiple conventional techniques failed Dramatic improvement in quality of life Restoration of nasal function and appearance	Two-stage approach using patient’s arm as bioreactor promoted integration in irradiated tissue Limited donor site morbidity 3D-printed porous structure allowed for tissue ingrowth	2023	Dupret-Bories [41]
Semi nasal reconstruction	3D scan	Unknown	forehead flap surgical guide								2024	Park [42]
Nasal stent prosthesis	Extraoral scanning and photogrammetry	PLA	Nasal stent								2024	Rathee [43]
Open rhinoplasty in chondrosarcoma case	CT and MRI scanning	3D-printed bone model	Implantable structures								2024	Yamamoto [44]

**Table 4:** Comparative analysis matrix of conventional vs. 3D-printing approaches in nasal reconstruction.

Parameter	Conventional Techniques	3D-Printing Approaches	Key Differentiating Factors
**Preoperative Planning**	Relies on 2D imaging and surgeon experience; limited ability to simulate outcomes	Patient-specific 3D models; virtual surgical planning; outcome simulation	3D approaches provide tangible models for planning and patient education; conventional approaches rely more heavily on surgeon experience
**Structural Accuracy**	Dependent on intraoperative judgment; may require multiple adjustments	Precise based on preoperative imaging; may not adapt to intraoperative findings	3D approaches offer higher initial precision but less adaptability; technical limitations in imaging-to-printing fidelity remain
**Tissue Compatibility**	Well-established biological integration of autologous tissues	Variable based on material selection; promising but limited long-term data	Conventional approaches have stronger evidence for long-term integration; 3D approaches still addressing biocompatibility challenges
**Surgical Learning Curve**	Steep learning curve; highly dependent on surgical expertise	Initial technical learning curve; potentially reduces reliance on advanced surgical skills	3D approaches may standardize certain procedures but require new technical competencies
**Resource Requirements**	Lower initial technology costs; higher operating room time	Higher technology/equipment costs; potentially reduced OR time	Cost–benefit analysis remains unclear; depends on healthcare system infrastructure
**Customization Capability**	Limited by available donor tissue; reliant on intraoperative modification	Highly customizable preoperatively; less adaptable intraoperatively	3D approaches offer greater preoperative customization but may struggle with intraoperative adjustments
**Clinical Evidence Base**	Extensive long-term outcomes data; well-documented complications	Limited long-term data; mostly case reports and small series	Conventional approaches have significantly stronger evidence base, particularly for Level 1 outcomes

**Table 5:** Hierarchical analysis of 3D printing applications in nasal reconstruction.

Application	Level 3 (Technical)	Level 2 (Surgical)	Level 1 (Clinical)	Evidence Quality
**Preoperative modeling**	High-accuracy anatomical models (±0.2 mm)	Reduced planning time (20–30%); improved surgical precision	Limited evidence for reduced complication rates; some evidence for improved aesthetic outcomes	Moderate
**Surgical guides**	Precise cutting and positioning guides (±0.3 mm)	Reduced operative time (15–30%); more accurate implementation of surgical plan	Emerging evidence for improved symmetry; limited evidence for reduced revision rates	Moderate–Low
**Patient-specific implants**	Exact anatomical fit (±0.5 mm); customized mechanical properties	Reduced need for intraoperative modification; simplified surgical technique	Isolated case reports of successful outcomes; no comparative studies with conventional techniques	Low
**Bioprinted scaffolds**	Controlled porosity and architecture; cell compatibility	Laboratory evidence of tissue integration	No clinical evidence of superior long-term outcomes	Very Low

## Data Availability

No new data were created as this paper is a review of the existing literature.
